# The Effect of Silver Diamine Fluoride Combined with Potassium Iodide and Sodium Fluoride on the Remineralisation of Hydroxyapatite

**DOI:** 10.3290/j.ohpd.c_1811

**Published:** 2025-01-23

**Authors:** Yan Ma, Haoran Chen, Yan He, Liming Tao

**Affiliations:** a Yan Ma Dentist, The Third Affiliated Hospital of Anhui Medical University, The First People’s Hospital of Hefei, Hefei, China. Conceptualisation, methodology, resources, data curation, validation, investigation, visualisation, formal analysis, wrote original draft, reviewed and edited the manuscript read and agreed to the published version of the manuscript.; b Haoran Chen Dentist, Queen Mary University of London Institute of Dentistry, London, UK. Conceptualisation, software, investigation, visualisation, wrote original draft, reviewed and edited the manuscript, read and agreed to the published version of the manuscript.; c; d Yan He Dentist, The Third Affiliated Hospital of Anhui Medical University, The First People’s Hospital of Hefei, Hefei, China. Conceptualisation, methodology, investigation, read and agreed to the published version of the manuscript.; e Liming Tao Dentist, Hehfei Stomatological Hospital, Hefei, China. Conceptualisation, validation, visualisation, resources, supervision, reviewed and edited the manuscript, read and agreed to the published version of the manuscript. †Ma and Chen contributed equally to this study.

**Keywords:** demineralisation, potassium iodide, remineralisation, silver diamine fluoride, sodium fluoride.

## Abstract

**Purpose:**

To compare remineralisation efficacy between silver diamine fluoride (SDF) combined with potassium iodide (KI) and sodium fluoride (NaF) varnish using hydroxyapatite (HAP) artificial white spot lesions (AWSLs) demineralisation model.

**Materials and Methods:**

A total of 25 HAP disks was randomly divided into five groups (n = 5): baseline, AWSLs, deionized water (DW), SDF-KI or F-varnish. After AWSLs were developed, the specimen was treated with either deionized water, SDF-KI or F-varnish. These specimens were then subjected to pH-cycling for 7 days. The remineralisation potential was assessed by measuring changes in Vickers hardness (VHN). Morphological and compositional analyses were conducted using scanning electron microscopy (SEM), energy dispersive x-ray (EDX), x-ray diffraction (XRD), and Fourier-transform infrared spectroscopy (FTIR). Ion-selective electrodes (ISE) were utilised to measure calcium and fluoride release.

**Results:**

SDF-KI treatment demonstrated statistically significant remineralisation potential in restoring VHN values vs baseline levels (p < 0.001). SEM, EDX, and XRD analyses confirmed the mineral deposits to indicate remineralisation. The uptake of calcium was higher in SDF-KI than in F-varnish (p = 0.011). The fluorapatite (FAP) and fluoride-substituted apatite formation were validated by FTIR and XRD analyses.

**Conclusion:**

SDF-KI and F-varnish applications are both effective in promoting remineralisation on HAP disks. The application of SDF-KI affected the physicochemical and mechanical properties of demineralised HAP. The SDF-KI showed more formation of fluoride-substituted apatite and is effective in the hardening of demineralised HAP.

White spot lesions (WSLs) are a common adverse effect of orthodontic treatment and can have lasting negative effects on dental aesthetics.^14^ Directly bonded orthodontic brackets and bands are commonly used in malocclusion treatment. However, this has resulted in poor oral hygiene.^7^ The WSLs are defined as enamel surface and/or subsurface demineralisation without cavitation.^40^ The prevalence of WSLs has risen by 29.2% between 1986 and 2012.^32^ It was reported that the prevalence of WSLs after orthodontic treatment increased from 5% to 97%.^13^ WSLs are likely to develop into carious cavities due to a lack of appropriate oral hygiene or remineralisation treatment. Topical fluoride is well-established in preventing demineralisation and managing caries. High doses of fluoride have been advised during and after orthodontic treatment to prevent the progression of WSLs into carious lesions.^16^ Apart from fluoride toothpastes, carriers such as gels,^25^ mouthrinses,^26^ and varnishes,^27^ can also deliver fluoride.

Fluoride varnish is an important strategy for WSL management. Most fluoride varnishes contain 22,600 ppm fluoride and have different compositions and delivery systems. This 5% NaF concentration is considered a cariostatic amount of fluoride.^46^ The dental varnish features delayed fluoride release, which increases its duration of contact with the tooth surface.^34^ This provides an intraoral reservoir of fluoride ions to hinder the cariogenic process over an extended duration, thereby better supporting the remineralisation process.^2,11^ Du et al^7^ demonstrated that fluoride varnish could be considered an efficient method for reversing WSLs and caries prevention after fixed orthodontic treatment. Shen et al^41^ then evaluated fluoride varnish in remineralisation, concluding that fluoride varnish is superior to varnish without fluoride in promoting remineralisation of WSLs.

Silver diamine fluoride (SDF) is used for the management of caries as a minimally invasive approach. SDF combines the remineralising effect of fluoride with the antimicrobial effect of silver, which promotes lesion arrest and reversal.^30^ The most common and efficacious working concentration of SDF is 38% (44,800 ppm fluoride). It is reported that SDF can penetrate enamel up to a depth of 25 µm with 2–3 times more fluoride retainment than NaF.^30^ SDF reacts with hydroxyapatite to produce calcium fluoride (CaF_2_) and silver phosphate (Ag_3_PO_4_), which increase the mineral density and microhardness of the carious lesions.^46^ An in-situ study also demonstrated that SDF can increase mineral density in early carious lesions.^31^ However, SDF application can give rise to esthetic concerns because of the release of free fluoride and silver ions. The Ag_3_PO_4_ precipitate causes black staining on the teeth.^33^ The application of KI (potassium iodide) can reduce this black staining^36^ by reacting with free silver ions. The yellow silver iodide precipitate is insoluble, which prevents black staining of teeth.^33,47^ Several studies have shown that the application of KI can reduce the side effects of SDF in carious lesions by reducing silver ions.^33,47^ Recent studies have reported that SDF with KI could promote remineralisation on artificial enamel lesions.^19,42^ However, the effect of remineralisation with respect to the physicochemical and mechanical properties of the treated WSLs should be further investigated. Therefore, the aim of this in-vitro study is to evaluate the remineralising effect of the combination of SDF and KI on the artificial WSLs.

## MATERIALS AND METHODS

### Sample Preparation

Twenty-five (25) HAP disks, 12 mm in diameter and 2 mm thick (Plasma Biotal; Buxton, UK), were varnished with nail polish (Maybelline; New York, NY, USA), leaving an unvarnished window of 3 mm x 4 mm on the surface of each disk. Five groups were used for the VHN test: baseline, AWSLs, SDF-KI, F-varnish and DW group. Each group included five HAP disks. Five HAP disks were selected as the baseline only. Artificial white spot lesions (AWSLs; n = 20) were then developed using demineralisation solution, while five HAP disks were left with AWSLs only. After that, 15 samples with AWSLs were allocated into three different treatment groups (SDF-KI, F-varnish and DW group), as shown in Table 1.

**Table 1:** The allocated test groups

Deionized water	Deionized water
SDF-KI (Riva Star, SDI; Bayswater, Australia)	38% silver fluoride
F-varnish (Duraphat, Colgate; Surrey, UK)	5% sodium fluoride

### Lesion Formation

To create the AWSLs on the HAP disks, each HAP disk was immersed in 10 ml of a demineralisation solution for 96 h. The demineralisation solution was prepared using 2.2 mM CaCl_2_, 2.2 mM KH_2_PO_4_, and 50 mM acetic acid. The pH was adjusted to 4.4 using 1M potassium hydroxide (KOH). The solutions were changed every day to avoid aggregation of demineralisation products and pH change. After 96 h, each specimen was washed thoroughly using DW.

### Treatment 

The samples from three different treated groups were dried using a three-in-one syringe, and a thin and uniform layer of dental varnish was applied to each HAP disk window according to the manufacturer’s instructions. The area was then gently wetted with DW to accelerate varnish setting.

### pH-Cycling

A standard pH-cycling regimen was used to mimic the pH dynamics of the oral environment. Specifically, each sample was first immersed in 10 ml of demineralisation solution for 3 h and then in remineralisation solution for 21 h each day for 7 consecutive days at 37°C. The pH-cycling solution was replaced every two days. The remineralisation solution was composed of 1.5 mM CaCl_2_, 0.9 mM KH_2_PO_4_, and 150 mM KCl, with the pH adjusted to 7.0 using 1M KOH.

### VHN (Vicker’s Hardness Number)

Following the development of AWSLs and 7 days of pH-cycling, Vicker’s hardness (VHN) was measured using a hardness tester (Vicker indenter Micromet II, Buehler; Lake Bluff, IL, USA) on a total of five samples from each group. Each sample was tested with an indentation load of 100 g for 15 s.^6^ The space between indentations was at least 50 μm to avoid interference and crack propagation in the central area of each sample. Ten indentations were made per sample. An average of 10 indentation readings for five samples was recorded.

### ISE (Ion-selective Electrodes)

Ca- and F-ISE were employed to measure Ca- and F-ion concentrations, respectively. The F-ISE (Nico2000; London, UK) was calibrated by gradient F^-^ concentration (serial dilutions of standard F^-^ solution: 1000 ppm, 100 ppm, 10 ppm, 1 ppm, 0.1 ppm) at room temperature. The Ca-ISE (Nico2000) was calibrated by gradient (4000 ppm, 400 ppm, 40 ppm, 4 ppm, 0.4 ppm). After calibration, Ca- and F-ion concentrations were measured for all groups at each time point in the 10 ml demineralisation and remineralisation solutions. The ISE calibration equation was provided by converting the ISE reading (mV) into concentration of F^-^ ions.

### FTIR (Fourier-Transform Infrared Spectroscopy)

The disks were pressed against the ATR-FTIR lens using a FTIR Spectrometer (Frontier, PerkinElmer; Waltham, MA, USA). The scans were undertaken in the range of 500–1500 cm^-1^ with a resolution of 4 cm^-1^ to collect the absorbance spectra. A background measurement was carried out before scanning the sample to ensure that all unwanted environmental effects were excluded from the analysis.

### SEM (Scanning Electron Microscopy)

The disks were observed in an SEM (FEI Inspect-F; Hillsboro, OR, USA) after sputter-coating with carbon (SC 7620, Quorum; Laughton, UK) for 45 s to maintain conductivity. Then the samples were analysed at an accelerating voltage of 5 kV and a spot size of 3.0 at a variable working distance (about 10 mm). After that, energy-dispersive x-ray (EDX) analysis was performed at a voltage of 20 kV with a spot size of 6.0.

### XRD (X-Ray Diffraction)

An X’Pert Pro X-ray diffractometer (Panalytical; Malvern, UK) emitting Cu-Kα alpha radiation scanned the samples using a 2θ range of 5 to 70 degrees in standard reflection mode, with sample holders spinning on the stage during the scans. The XRD analysis was carried out to determine the formation of apatite on the samples.

### Statistical Analysis

The Kruskal-Wallis H-test was applied to analyse VHN in all groups. The Mann-Whitney test was carried out for intergroup comparison in the VHN analysis. The Ca- and F-ion release in each group was analysed using Kruskal-Wallis H-tests with the Mann-Whitney test. The Ca:P atomic ratio was analysed using one-way ANOVA and Tukey’s post-hoc test. The F^-^ ratio was analysed using the Kruskal-Wallis H test with the Mann-Whitney test. A statistically significance level of 0.05 was set, and all tests were run using SPSS 29.0 (SPSS; Chicago, IL, USA).

## RESULTS

### VHN

**Fig 1:**
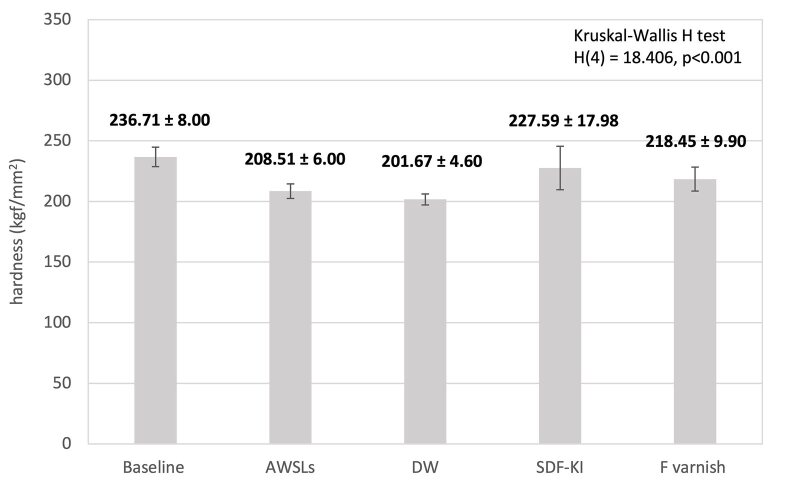
Mean VHN value (SE) of HAP disks at baseline, AWSLs and following the application of SDF-KI, F varnish and DW.

The mean Vicker’s hardness ( ± SE) of the samples is shown in Fig 1, ranging from 236.71 ± 8.00 (baseline) to 208.51 ± 6.00 (AWSLs). After treatment and 7 days of pH-cycling, the mean VHN value (SE) of HAP disks was 201.67 ± 4.60 (DW), 227.59 ± 17.98 (SDF-KI) and 218.45 ± 9.90 (F^-^ varnish), respectively. The Kruskal-Wallis H-test demonstrated statistically significant differences between all groups, H(4)=18.406; p < 0.001. The Mann-Whitney test revealed statistically significant differences between baseline and DW group (U = 619.000; p < 0.001); baseline and SDF-KI group (U = 787.000, p < 0.001); AWSLs and SDF-KI group (U = 953.000; p = 0.041); baseline and AWSLs (U = 901.000, p = 0.016). In all treatment groups, the VHN of SDF-KI is the highest, while the VNH of DW is the lowest.

### ISE

#### Fluoride ion release 

**Fig 2a to d:**
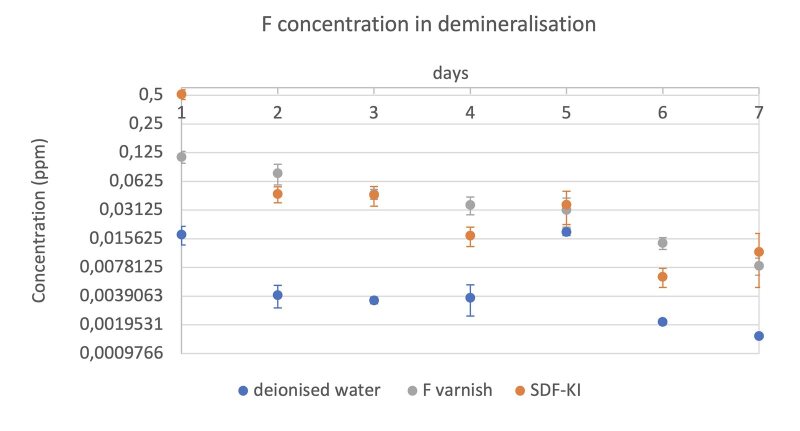

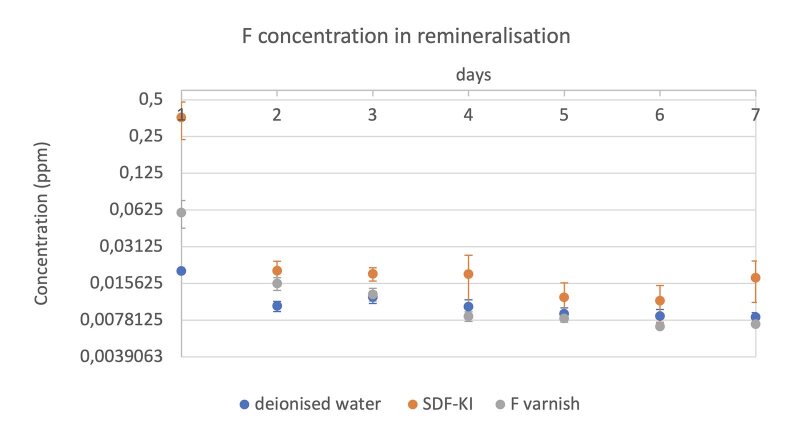

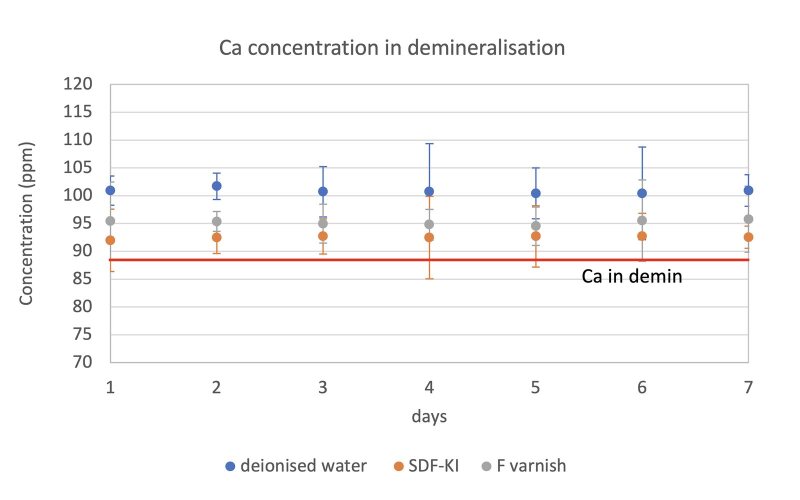

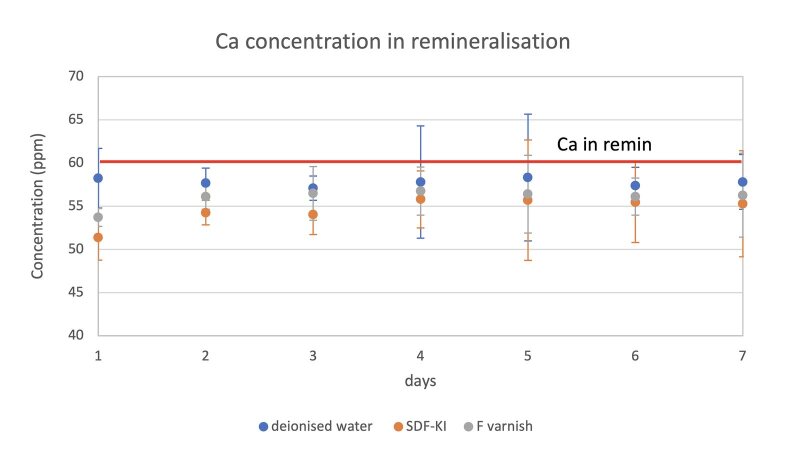
The F ion release profile of each treatment group immersed in pH 4.4 demineralisation (a) and pH 7 remineralisation (b) solution; Ca ion release profile of each treatment group immersed in pH 4.4 demineralisation (c) and pH 7 remineralisation (d) solution.

The F^-^ concentration decreased during the pH-cycling process (Figs 2a and 2b). The fluoride release in SDF-KI on the first day was higher than F-varnish in the demineralisation and remineralisation process. However, the F^-^ concentration was higher after day 2 in the demineralisation solution for the F-varnish group when compared to the SDF-KI varnish group. The F^-^ concentration could not be measured after day 1 in the remineralisation solution, as the limitation of the F-ISE technique is 0.02 ppm. Furthermore, there was no F^-^ detected in the DW group during pH cycling.

#### Calcium ion release

Figures 2c and 2d depict the concentration profiles of calcium in the pH-cycling process. The Ca concentration was 88 ppm and 60 ppm in the demineralisation and remineralisation solution, respectively. Figure 2c show that the loss of concentration of Ca in all groups increased from day 1 to day 7. The cumulative average concentration of Ca ions in demineralisation, remineralisation and combined is shown in Table 2. Regarding the remineralisation process (Fig 2d), the Ca concentration in SDF-KI group was the lowest, which demonstrated more uptake of Ca ions in the SDF-KI group. Furthermore, the DW group also showed uptake of Ca ions in the remineralisation phase.

**Table 2:** >The pH-cycling cumulative group averages (and statistical data) from the demineralisation, remineralisation, and combined periods

Demineralisation
Deionized water	0.01 ± 0.003	Kruskal-Wallis H (p = 0.014) Deionized water and SDF-KI (p = 0.018) Deionized water and F^-^ varnish (p = 0.009) SDF-KI and F^-^ varnish (p = 0.848)	100.83 ± 0.16	Kruskal-Wallis H (p < 0.001) Deionized water and SDF-KI (p = 0.002) Deionized water and F^-^varnish (p = 0.002) SDF-KI and F^-^ varnish (p = 0.002)
SDF-KI	0.10 ± 0.07	92.52 ± 0.10
F^-^ varnish	0.05 ± 0.01	95.21 ± 0.18
Remineralisation
Deionized water	0.01 ± 0.002	Kruskal-Wallis H (p = 0.047) Deionized water and SDF-KI (p = 0.018) Deionized water and F^-^ varnish (p = 0.565) SDF-KI and F^-^ varnish (p = 0.064)	57.74 ± 0.17	Kruskal-Wallis H (p < 0.001) Deionized water and SDF-KI (p = 0.002) Deionized water and F^-^ varnish (p = 0.002) SDF-KI and F^-^ varnish (p = 0.018)
SDF-KI	0.07 ± 0.05	54.54 ± 0.59
F^-^ varnish	0.02 ± 0.007	55.96 ± 0.39
Combined demineralisation and remineralisation
Deionized water	0.02 ± 0.003	Kruskal-Wallis H (p = 0.011) Deionized water and SDF-KI (p = 0.009) Deionized water and F^-^ varnish (p = 0.013) SDF-KI and F^-^ varnish (p = 0.655)	10.57 ± 0.23	Kruskal-Wallis H (p < 0.001) Deionized water and SDF-KI (p = 0.002) Deionized water and F^-^ varnish (p = 0.002) SDF-KI and F^-^ varnish (p = 0.002)
SDF-KI	0.17 ± 0.12	-0.94 ± 0.67
F^-^ varnish	0.06 ± 0.02	3.17 ± 0.36

### FTIR 

**Fig 3a to c:**
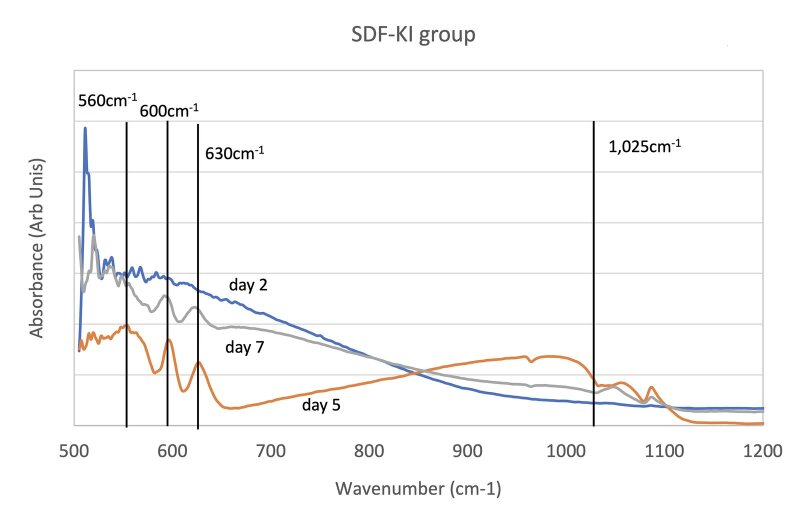

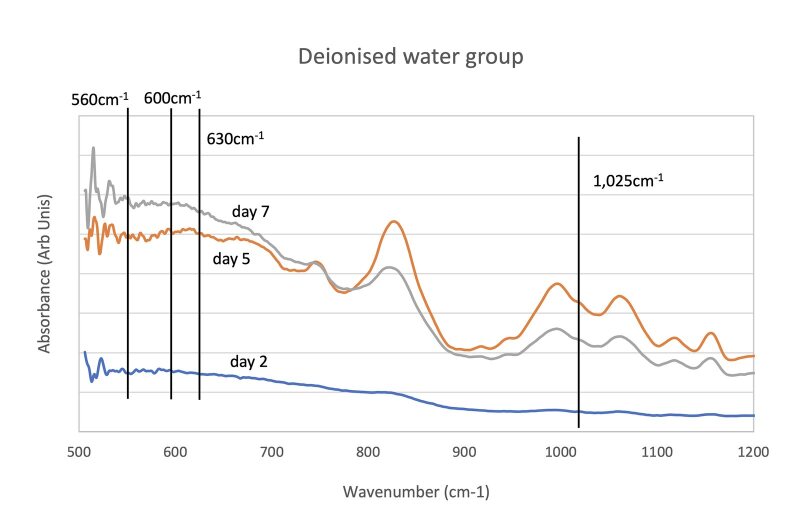

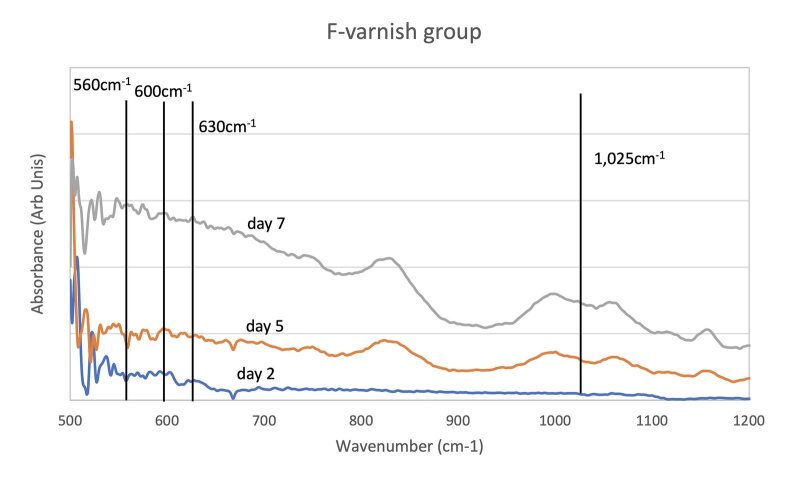
FTIR spectra of HAP treated with DW (a), SDF-KI (b), F- varnish (c).

Figure 3 illustrates the FTIR spectra of the samples at days 2, 5 and 7 of pH cycling. For HAP, the PO_4_^3^^−^ band appears at ~ 560 and ~ 600 cm^-1^.^5^ Suchanek et al^43^ showed an OH vibration mode of about 630 cm^-1^ for HAP.^43^ These peaks were not present in the DW group. In the SDF-KI group, these peaks were highest on day 5. In the F-varnish group, there was a small peak at 600 cm^-1^, and the peak was also the highest on day 5. It is noted that the peak at 630 cm^-1^ increased from day 2 to day 7 in the F-varnish group.

### SEM

**Fig 4a to d:**
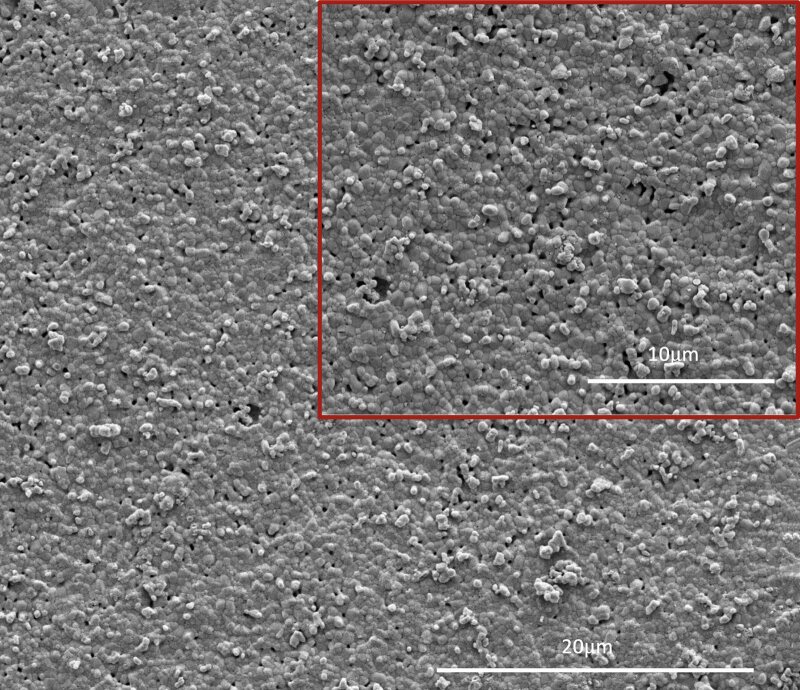

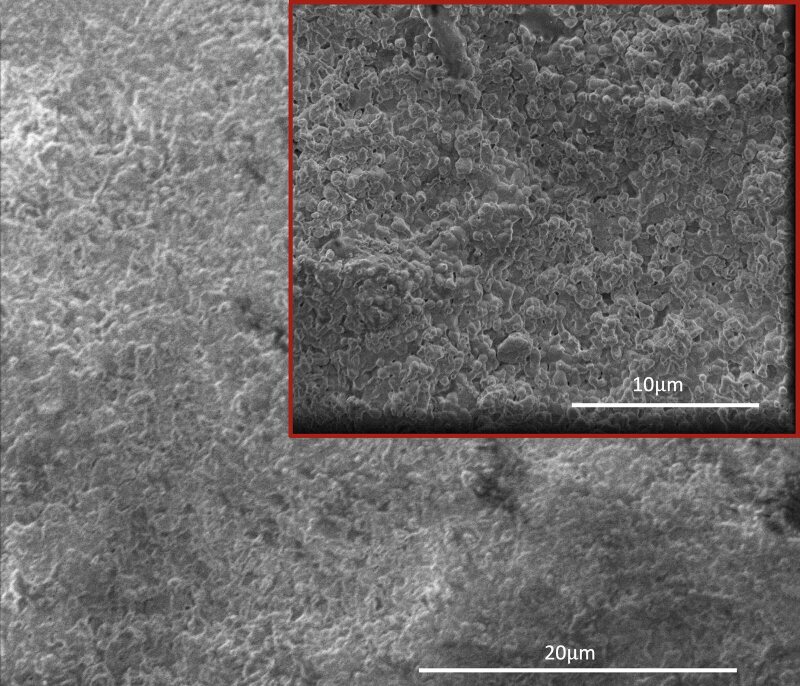

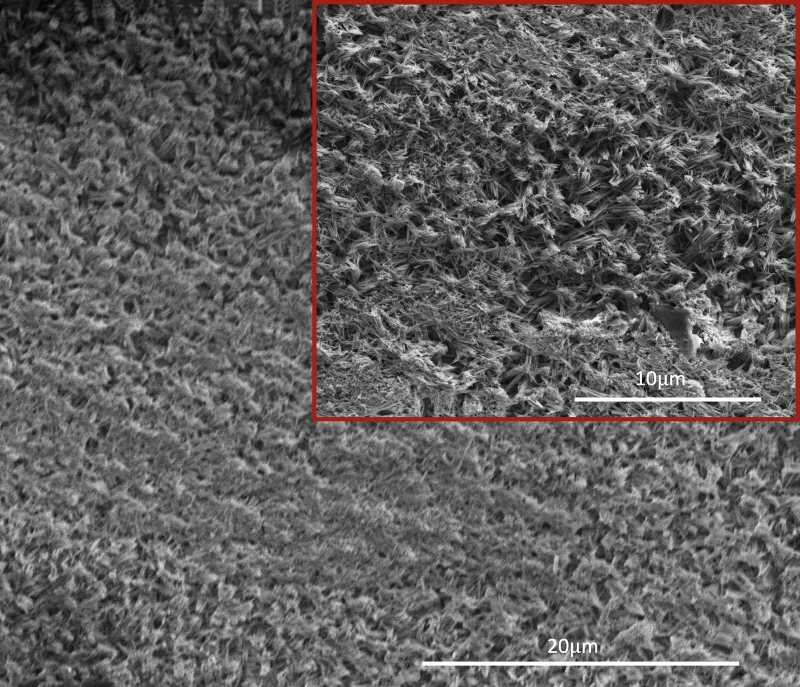

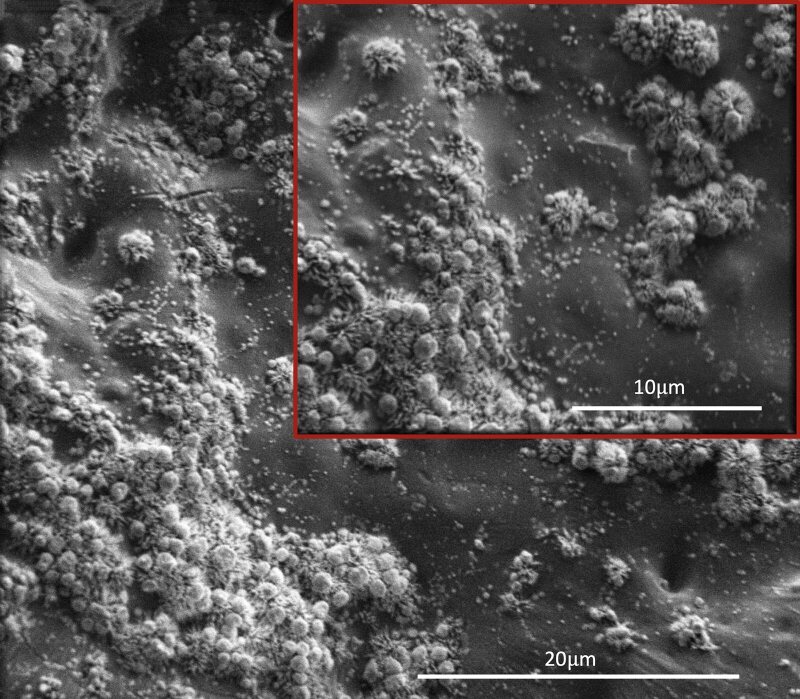
SEM images (5000 and 10000X) of each sample in AWSLs (a), after pH-cycling (b) treated with DW, (c) SDF-KI, and (d) F- varnish.

The SEM images of different treatments were collected after 7 days of pH cycling. Properties such as surface morphology and composition of the sample surface were examined using this technique. Figure 4b shows uneven and porous HAP-disk surfaces treated with DW and pH cycling. SEM images after SDF-KI application revealed sharp, acicular, and irregular particles (Fig 4c). Regarding the F-varnish group, the samples showed globular, irregular, and acicular particles at the AWSLs sites (Fig 4d).

The results of EDX element analysis comparing the Ca, P, F and Ca:P ratio in AWSLs and three treatment groups are shown in Table 3. In the AWSLs group, the Ca:P ratio was 1.54 and 1.55 in the DW group. The Ca:P ratios were 1.69 and 1.71 for SDF-KI and F-varnish groups, respectively. This means that mineral deposits were observed in two varnish groups after pH cycling (p = 0.005). The element F (fluorine) in the F-varnish group was statistically significantly higher than that in the SDF-KI group (p = 0.021). Interestingly, fluorine was detected in the DW group, although there was no fluoride exposure.

**Table 3:** Ca, P, F and Ca:P ratio (atomic%) in AWSLs and three treatment groups for HAP disks (5000X), (statistical analysis for Ca:P ratio)

AWSLs	Ca	15.49 ± 1.20	One-way AVOVA for Ca:P ratio p = 0.005 Multiple comparison: DW and SDF-KI (p = 0.010) DW and F^-^ varnish (p = 0.004) DW and AWSLs (p = 0.962) SDF-KI and F^-^ varnish (p = 0.642) AWSLs and SDF-KI (p = 0.010) AWSLs and F^-^ varnish (p = 0.004). Kruskal-Wallis H-test for fluoride p = 0.007 Mann-Whitney U-test: DW and SDF-KI (p = 0.021) DW and F^-^ varnish (p = 0.021) SDF-KI and F^-^ varnish (p = 0.021).
P	10.02 ± 0.69
F	0.09 ± 0.01
Ca/P	1.54 ± 0.01
Deionized water	Ca	14.91 ± 0.36
P	9.64 ± 0.21
F	0.15 ± 0.07
Ca/P	1.55 ± 0.02
SDF-KI	Ca	17.62 ± 0.12
P	10.43 ± 0.13
F	5.45 ± 0.11
Ca/P	1.69 ± 0.03
F-varnish	Ca	9.18 ± 0.60
P	5.40 ± 0.47
F	32.88 ± 1.56
Ca/P	1.71 ± 0.05

### XRD

**Fig 5:**
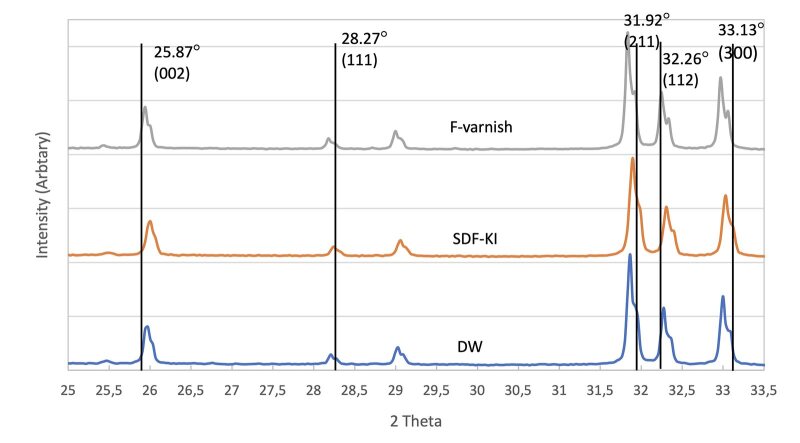
XRD spectrum of HAP treated with DW, treated with SDF-KI, treated with F- varnish.

Figure 5 depicts the XRD patterns of FAP (ICDD: 05-015-0876) as a black line with the four most intense peaks at 25.87 degrees (d = 3.44 Å, plane 002), 31.92 degrees (d = 2.80 Å, plane 211), 32.26 degrees (d = 2.77 Å, plane 112), and 33.13 degrees (d = 2.70 Å, plane 300). The F-varnish group showed two sharp peaks at 31.92 and 32.26 degrees, respectively. The XRD pattern in the SDF-KI group revealed a sharp diffraction line approaching 31.92 degrees, and a small, broad peak at 33.13 degrees, which confirmed the presence of more crystalline apatite. A broad diffraction peak in the DW group also was present at 31.92 degrees.

## DISCUSSION

This laboratory-based study aimed to compare the effects of SDF-KI and NaF varnish on AWSLs. The enamel is composed of 96% HAP. In this study, HAP disks were utilised to simulate the demineralisation of human enamel resulting in WSLs, as this method can avoid the variation from tooth to tooth in vivo. VHN is a minimally destructive technique that has been frequently used to evaluate remineralisation on the enamel surface.^28,38,39^ VHN values of AWSLs and treatment groups were statistically significantly lower than that of the baseline group, due to mineral loss from the HAP disks. The results of Ca-ISE in the demineralisation phase support the loss of calcium. This indicated that uniform AWSLs were obtained using the demineralisation solution. VHN values showed no statistically significant difference between the DW and AWSL group. This could be attributed to the lack of additional F^-^ ions to form apatite on the demineralised HAP. SDF-KI and F-varnish showed greater remineralisation as a result of mineral deposition within AWSLs, as reflected in higher VHN. The application of SDF-KI restored the VHN values to the baseline level. This is supported by a microCT study which showed an increased mineral density after the application SDF-KI and F varnish.^19^ This may explain the increase in VHN. Sorkhdini et al^42^ found that the VHN value of SDF-KI is higher than that of F varnish on artificial enamel lesions after five days of pH cycling (p < 0.0001). However, those authors also showed that the VHN value of SDF is highest among SDF, SDF-KI and F varnish groups under the same conditions (p < 0.0001).^42^ Farhadian et al^10^ reported that carious lesions treated with SDF have the same hardness values as sound enamel; in that study, it is noteworthy that the enamel lesions treated with SDF were stored in artificial saliva. The main mechanism of SDF to increase hardness can be explained by the formation of Ag_3_PO_4_, CaF_2_, and FAP on treated lesions.^24^ In this study, pH-cycling employed a pH-4.4 demineralisation solution and a pH-7 remineralisation solution. FAP is dissolved at a critical pH of 4.5, which decreases the VHN value. This was supported by the SEM results which showed porous areas and voids of the demineralised HAP for all groups exposed to the demineralisation solution. In addition, the ISE results after demineralisation confirmed the dissolution of FAP to release F^-^ ions. This explains the existence of two similar F^-^ concentrations in the demineralisation solution for both SDF-KI and F-varnish groups from day 2. However, the ISE technique is unable to measure concentrations of F^-^ < 0.02 ppm,^4^ which was the case between days 6 and 7 during demineralisation.

The F^-^ concentration in SDF (44,000 ppm) is much higher than F-varnish (22,000 ppm). Therefore, the maximum fluoride ion release was observed with SDF-KI followed by F-varnish on day 1. A low concentration of fluoride in the oral cavity can react with dissolved hydroxyapatite when the pH falls below the critical pH. Mohammed et al^29^ reported fluoride-substituted apatite formation at a concentration of 45 ppm fluoride, while higher concentrations resulted in CaF_2_ formation.^29^ XRD confirmed the fluoride-substituted apatite and CaF_2_ formation at 28.27 degrees 2θ corresponding to the 111 plane.^21^ The results of SEM and EDX also supported CaF_2_ and fluoride-substituted apatite formation. The globular^22^ and needle-like^15^ crystal precipitated on the HAP surface in the F-varnish group. CaF_2_ is insoluble in saliva at neutral pH, so it can stick to the tooth surface for weeks or months after topical fluoride application. CaF_2_ is considered a reservoir of fluoride during cariogenic challenges. At cariogenic pHs, the adsorption of primary phosphate (H_2_PO_4_^-^) to calcium sites was unable to inhibit the dissolution of CaF_2_.^37^ EDX measured high fluorine concentrations in the SDF-KI and F-varnish groups. It is noted that SEM and EDX are superficial techniques, and many acicular precipitates in the SDF-KI group may have the appearance of KI.^23^ This could explain why the fluorine ratio of SDF-KI is lower than that of F-varnish. In addition, fluorapatite and CaF_2_ cannot dissolve in a pH-7 solution, so that no fluoride was detected without free fluoride release from day 2 in the remineralisation solution.

An uptake of Ca was observed in the DW group in remineralisation solution. Chen et al^4^ also reported Ca uptake after DW treatment in the pH-7 remineralisation solution. In terms of the pH-7 remineralisation solution containing CaCl_2_, KH_2_PO_4_, KCl and KOH buffer, these components may affect remineralisation. Ionta et al^20^ indicated that pH-7 artificial saliva can promote partial enamel remineralisation. Their artificial saliva remineralisation solution consisted of 0.7mM CaCl_2_·2H_2_O, 0.4 mM MgCl_2_, 4 mM KH_2_PO_4_, 20_2_mM Hepes buffer acid form, 30mM KCl in 1000 ml distilled water (pH 7).^20^

FTIR spectra of HAP exhibited characteristic peaks at approximately 561 cm^−1^, 601 cm^−1^, and 1024 cm^−1^, which are attributed to the stretching vibration bands of the phosphate group. The 1024 cm^−1^ peak is v3 PO_4_^3-^, and Beniash et al^3^ reported a broad peak with a maximum of 1115 cm^−1^ and a small hump with a maximum of 1025 cm^−1^ in the outer layer of enamel. Gadaleta et al^12^ described a similarly shaped ν3 peak in synthetic amorphous calcium phosphate, which is a precursor of HAP.^12^ The FTIR data suggested that the SDF-KI formed more apatite, as indicated by the PO_4_^3-^ peak at 600 cm^-1^. A study by Chen et al^5^ showed a v4 (PO_4_^3-^) band at 600 cm^-1^ for HAP and FAP. HAP and FAP belong to a large group of calcium apatites with the formula Ca_10_(PO_4_)_6_X_2_. If X describes hydroxyl groups, the mineral is called HAP (Ca_10_(PO_4_)6(OH)_2_), and, in the case of F ions, F-HAP (Ca_10_(PO_4_)_6_(OH,F)_2_) is a result of hydroxyl ions being substituted (at least partly) by fluoride.^35^ This could explain the formation of either fluoride-substituted apatite or fluorapatite. It is difficult to distinguish HAP from FAP using the FTIR. The results from XRD supported the findings of fluoride-substituted apatite formation. The 002 peak is overlapped by FAP and HAP crystals. Regarding the diffraction lines between 31 degrees and 33.5 degrees 2θ, the reflection peaks 211 and 300 of FAP are shifted toward a higher 2θ compared to that of HAP and fluoride-substituted apatite.^44^ This study found that the 211 peak of SDF-KI was shifted to a slightly lower angle, meaning the formation of fluoride-substituted apatite. Furthermore, the low peak intensity indicated a small amount of apatite, and the broad line was related to a small crystallite size. The atomic Ca:P ratio for HAP or FAP synthesis was 1.67, representing remineralisation in the HAP disks.^8^ If the Ca:P molar ratio is higher than 1.67, excess Ca causes the formation of CaO; if the Ca:P molar ratio is less than 1.67, excess P also causes the CaF_2_ phase.^9^ In addition, a high Ca:P ratio may be found in carbonate-apatites, or apatites containing additional calcium as CaO or hydroxide impurities.^44^ This study confirmed fluoride-substituted apatite formation after SDF-KI and F-varnish. For a future study, 19F Magic angle spinning-nuclear magnetic resonance spectroscopy (19F MAS-NMR) will be used to determine the F% in apatites. However, it should be noted that HAP differs from human enamel. The main difference between human enamel minerals and HAP is the presence of approximately 3 wt% carbonate in human enamel,^45^ which results in its faster dissolution compared to HAP disks.^1^ Due to their homogeneous chemical composition and structure in contrast to human enamel minerals, HAP disks have been acknowledged as appropriate analogues for dental enamel minerals.^17,18.^

## CONCLUSION

The use of SDF-KI and F-varnish are both effective in promoting remineralisation on HAP disks. The application of these products causes the precipitation of different crystalline salts, which increases the mechanical properties of demineralised HAP. The SDF-KI showed more formation of fluoride-substituted apatite and is effective in the hardening of demineralised HAP.
